# Coping with the burden of irritable bowel syndrome by emotional suppression—A cross sectional observational pilot study

**DOI:** 10.1002/jgf2.70060

**Published:** 2025-09-03

**Authors:** Henning Sommermeyer, Magdalena Ciesla, Dominika Szczerbiec, Pawel Olszewski, Paulina Wojtyla‐Buciora, Jacek Piatek

**Affiliations:** ^1^ Department of Health Sciences Calisia University Kalisz Poland

**Keywords:** Anger, anxiety, Courtauld Emotional Control Scale, depression, emotional suppression, irritable bowel syndrome

## Abstract

**Background:**

Psychological stress like depression, anxiety, and anger is a common comorbidity of irritable bowel syndrome (IBS). This clinical pilot study aimed to investigate if individuals employ emotional suppression to cope with the psychological burden associated with IBS.

**Methods:**

Emotional suppression was measured with the Courtauld Emotional Control Scale (CECS) in non‐IBS (50 women/50 men; average age 24.5 years) and IBS subjects (58 women/41 men; average age 41.0 years). IBS diagnosis was performed using the IBS questionnaire for Health Care Providers of the World Gastroenterology Organization, and the severity of IBS symptoms was assessed with the IBS Severity Scoring System.

**Results:**

Individuals with moderate to severe IBS showed significantly higher emotional suppression compared with non‐IBS subjects. Scores (median, interquartile range (IQR)) of the general coefficient of emotional control were 52.0 (IQR 48–56) vs. 45.0 (IQR 43–48) (*p* < 0.001), for depression 19.0 (IQR 17–21) versus 15.5 (IQR 15–17) (*p* < 0.001), for anxiety 17.0 (IQR 15–18) versus 15.0 (IQR 14–16) (*p* < 0.001), and for anger 16.0 (IQR 15–19) versus 14.5 (IQR 13–16) (*p* < 0.001) for IBS and non‐IBS subjects, respectively.

**Conclusion:**

IBS patients have higher emotional control levels compared with non‐IBS patients. Concealing emotions is considered a negative approach for dealing with psychological stress. Physicians and individuals with IBS should be aware of these facts. Including therapeutic approaches to address emotional control as part of IBS treatment is recommended.

## 1 INTRODUCTION

Irritable bowel syndrome (IBS) is a disorder of the gut–brain interaction characterized by recurrent abdominal pain, bloating, and alterations in stool form and frequency.^1^ At present, there is no reliable biological marker supporting the diagnosis of IBS. Consequently, the diagnosis is solely based on symptom‐based criteria, after excluding organic gastrointestinal diseases.^2^, ^3^, ^4^ Published prevalence rates for IBS vary significantly, which is assumed to be caused by the diagnostic criteria used and differences in geography, culture, and populations studied.^5^ According to the Rome Foundation Global study, the overall prevalence of IBS ranges from 3.8% (Rome IV criteria) to 10.1% (Rome III criteria).^6^ IBS is diagnosed more frequently in women than in men and manifests during adolescence.^7^ The cause of the disease is still not fully understood and is assumed to be multifactorial, with environmental, inherited, and psychosocial factors contributing. Several potential mechanisms are discussed, among them visceral hypersensitivity, a disturbed function of the gut epithelial barrier, altered gastrointestinal motility, alterations of the enteroendocrine signaling, overactivity of the immune system, dysfunction of the gut–brain axis, and dysbiosis of the gut microbiota.^8^, ^9^ While IBS is not fatal, the disease troubles individuals because of the unpredictability of symptoms. Subjects with IBS experience negative impacts on their quality of life and work productivity.^10^, ^11^

IBS is frequently associated with a high prevalence of psychological disorders, with depression and anxiety being the most commonly diagnosed comorbidities in affected individuals.^12^, ^13^, ^14^, ^15^, ^16^, ^17^, ^18^ Elevated levels of depression and anxiety compared to those in healthy controls have been found in several studies.^19^, ^20^, ^21^ In about one‐fifth of IBS subjects, depression and anxiety are co‐occurring comorbidities.^22^ In contrast to depression and anxiety, anger in IBS subjects has been less intensively researched but is assumed to be a regular comorbidity as well.^14^, ^16^, ^23^

There seems to be a bidirectional link (the gut–brain axis) between the gastrointestinal symptoms of IBS and its psychological comorbidities. Clinical studies found that individuals with symptoms of depression and anxiety with no comorbid IBS developed gastrointestinal symptoms over time, whereas those with a diagnosis of IBS but no depression or anxiety at baseline developed symptoms of depression or anxiety.^24^, ^25^ Interestingly enough, in a majority of individuals, the gut symptoms precede the development of psychological stress.^25^ IBS severity increases significantly with the number of different psychological comorbidities (e.g., depression, anxiety, somatic symptom disorder, perceived stress, and gastrointestinal symptom‐specific anxiety).^22^, ^26^, ^27^

Coping strategies are psychological processes consciously used by individuals as a response to stress, anxiety, or difficult situations. Emotional suppression is a coping strategy where individuals inhibit the outward expression of emotions as a reaction to psychological stress. So far, little is known about emotional suppression in individuals suffering from IBS. Many subjects consider depression, anxiety, or anger uncomfortable and problematic. For some diseases (e.g., cancer, COVID‐19 infections) and medical situations (e.g., learning about a breast cancer diagnosis), it has been found that individuals respond to the associated psychological stress (e.g., depression, anxiety, and anger) by suppressing (controlling) their emotions.^28^, ^29^, ^30^ Emotional suppression is a semi‐conscious or unconscious coping strategy that avoids negative emotions. In general, emotional suppression is considered problematic. Expressing negative emotions is a beneficial practice that is recommended in many forms of psychotherapy, as their long‐term suppression may become the basis of many psychosomatic disorders.^31^, ^32^

Little is known if IBS subjects employ emotional suppression as an avoidance‐coping strategy for the psychological stress associated with their disease. To our knowledge, there is only one study with four participants that investigated emotional suppression in IBS subjects.^33^ In that particular study, emotional suppression was observed in one of the four individuals investigated. If this proportion (1 out of 4) is higher than non‐IBS individuals remained unclear, as the study investigated only IBS subjects. The Courtauld Emotional Control Scale (CECS) has been established for the assessment of emotional suppression in clinical trials.^34^ The CECS scale is a questionnaire with 21 statements measuring suppression of depression, anxiety, and anger feelings with three subscales, each comprising seven questions. Combining the results from the three subscales generates a general coefficient of emotional control. The present clinical pilot study used the CECS to compare the level of emotional control in IBS subjects with that of non‐IBS subjects.

## 2 METHODS

### 2.1 Study design

The study was advertised to individuals visiting the family doctor's clinic in 62‐820 Stawiszyn, Poland. Interested individuals were informed about the details of the study by their physicians. Individuals willing to participate were asked to sign an informed consent. Those who signed the informed consent were enrolled in the study by their physicians. The study was performed between March and August 2023. Individuals were diagnosed with the IBS questionnaire for health care providers (HCP) developed by the World Gastroenterology Organization (WGO) based on the Rome III diagnostic criteria for IBS.^35^ The IBS questionnaire evaluates individuals by asking patients about the presence and changes of abdominal pain or discomfort during the last 3 and 6 months. It evaluates if abdominal pain or discomfort is related to defecation and if it is associated with changes in stool frequency and/or changes in stool form. In addition, feelings of incomplete stool evacuation, urgency for going to the toilet, bloating, and issues with gas are assessed. Depending on the answers selected, a total score is calculated, which is then used to establish the presence or absence of IBS. The aim was to enroll 100 individuals diagnosed positively for moderate to severe IBS (the IBS group) and 100 individuals with a negative IBS diagnosis (the non‐IBS group). Neither individuals nor physicians were receiving an incentive for their participation in the trial. The study protocol was approved by the Ethics Committee of Calisia University (project identification code 1/2023). The trial was conducted following the Declaration of Helsinki. Informed consent was obtained from all subjects involved in the study. This research received no external funding.

### 2.2 Study participants, diagnosis of IBS, and assessment of severity of IBS

The study recruited women and men individuals aged 18–65 years. Subjects diagnosed with IBS (IBS questionnaire for HCP scores between 15 and 24 points) were checked to see whether they met exclusion criteria. Exclusion criteria comprised the use of products containing probiotic bacteria or treatment with antibiotics within the last 3 months, concurrent severe illness (malignancies, uncontrolled hypertension or diabetes, hepatic, renal, or cardiac dysfunctions, serious neurological disorders, psychosis, respiratory disorders such as asthma or chronic obstructive pulmonary disease, and hyper‐ or hypothyroidism), chronic bowel disorders other than IBS, including inflammatory bowel disease, gastroenteritis, stomach and duodenal cancer, celiac disease, pregnancy or lactation, diagnosed lactose intolerance, use of motility drugs or dietary fiber supplements within the last 2 weeks, taking anticoagulation medication, and participating in a clinical trial within the past 3 months. IBS subjects not excluded were then assessed for the severity of their IBS by using the IBS‐Severity Scoring System (IBS‐SSS).^2^ Only IBS subjects with moderate or severe IBS (IBS‐SSS scores ≥ 175) were included in the IBS group. Individuals assessed to have IBS questionnaire scores below a score of 15 and not meeting any of the study exclusion criteria were assigned to the non‐IBS group.

### 2.3 Assessment of emotional suppression

Emotional suppression was assessed by employing the Courtauld Emotional Control Scale (CECS) in the version compatible with the Polish adaptation by Juczynski.^34^, ^36^ The CECS comprises 21 statements divided into three subscales. Each of the subscales contains seven statements that concern the manner of showing depression, anxiety, and anger. Each subscale statement starts with “When I feel…” followed by either “unhappy (miserable),” “afraid (worried),” or “angry (very annoyed).” In the depression subscale, this is followed by: (1) “I bottle it up,” (2) “I let others see how I feel,” (3) “I keep quiet,” (4) “I hide my unhappiness,” (5) “I smother my feelings,” (6) “I refuse to say anything about it,” and (7) “I put on a bold face.” Statements in the anxiety subscale are: (1) “I tell others all about it,” (2) “I let others see how I feel,” (3) “I refuse to say anything about it,” (4) “I say what I feel,” (5) “I bottle it up,” (6) “I keep quiet,” and (7) “I smother my feelings.” The anger subscale comprises the following seven statements: (1) “I keep quiet,” (2) “I smother my feelings,” (3) “I hide my annoyance,” (4) “I bottle it up,” (5) “I say what I feel,” (6) “I refuse to argue or say anything,” and (7) “I avoid making a scene.” The CECS is designed to test adults, both healthy ones and sick individuals. The scale serves to measure respondents' control of depression, anxiety, and anger in difficult life situations. By marking the most suitable answer, respondents assess how often they express emotions in a way provided in the questionnaire on a four‐point scale from “almost never”—one point to “almost always”—four points. For each of the subscales, the results are calculated separately. The sum of the results in each subscale ranges from 7 to 28 points. A general emotional control coefficient is calculated by adding together the scores of the three subscales. The general emotional control coefficient can range from 21 to 84 points. The higher the score, the more suppressed emotions are. The reliability (Cronbach's alpha) of the Polish version of the CECS is 0.77 for depression control, 078 for anxiety control, 0.80 for anger control, and 0.87 for the general coefficient of emotional control (CECS).^36^

### 2.4 Statistical analysis

Data for age, weight, height, body mass index (BMI), and the IBS Questionnaire for HCP of WGO in both groups (non‐IBS and IBS group) were tested for normal distribution using the Kolmogorov–Smirnov normality test. To evaluate the associations between gender (women/men) in both groups, the Pearson's Chi‐squared test was performed. The Mann–Whitney *U* test was used for data that did not follow a normal distribution to compare the medians of the two study groups: the non‐IBS group and the IBS group. For data that followed a normal distribution, such as BMI between the non‐IBS and the IBS groups, Student's *t*‐test was performed. All calculations were performed using Statistica software version 14.1.0.4 (Tibco Software Inc., Palo Alto, CA, USA). The results were considered statistically significant at *p*‐values at least <0.05.

## 3 RESULTS

### 3.1 Participant flow, study progress, and baseline characteristics of the non‐IBS and IBS groups

**FIGURE 1:**
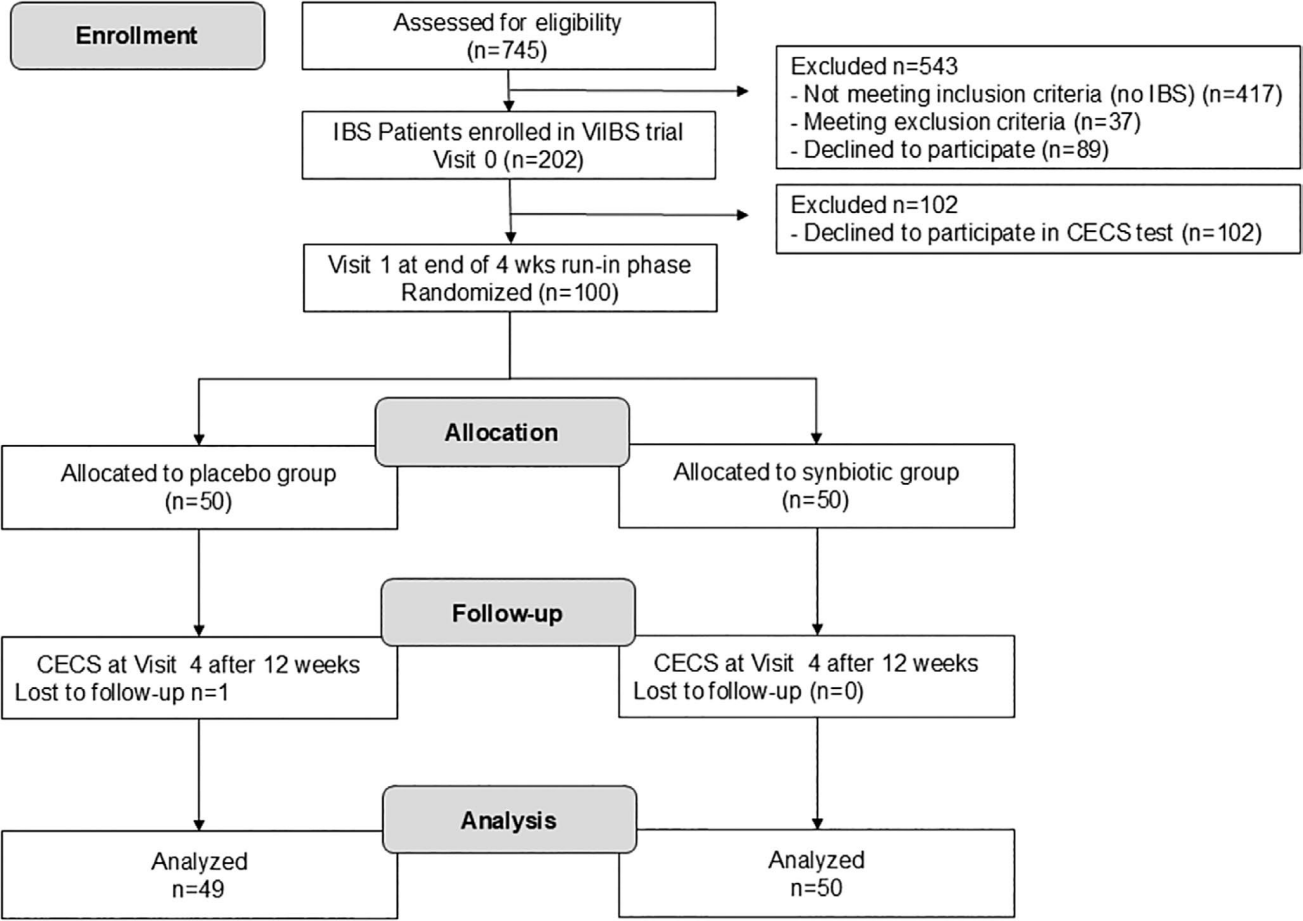
Participant enrollment and study progress.

Figure 1 shows the flow of the individuals through the trial. A total of 845 individuals were assessed for the possibility of participating in the study. Of these, 191 patients declined to participate in the trial. The remaining 654 were evaluated with the IBS questionnaire for HCP of the WGO. A total of 137 individuals were diagnosed with IBS. In these individuals, the IBS severity was assessed with the IBS‐SSS. The number of individuals that had to be excluded because of meeting exclusion criteria or failing to meet the inclusion criteria of an IBS‐SSS score ≥175 was 37. The emotional control level was assessed in the remaining 100 IBS individuals with the CECS scale. Data from one IBS trial participant had to be excluded from the final analysis as the participant's data set turned out to be incomplete. Of the total 517 identified non‐IBS individuals who agreed to participate in the trial, meeting the inclusion criteria and none of the exclusion criteria, the first 100 were enrolled in the study. No matching of a gender ratio or other demographic variables was considered. In hindsight, this proved to be a mistake, as the average age in the non‐IBS group was significantly lower than that in the IBS group. Emotional control levels were assessed in these individuals using the CECS scale. For the final analysis, data from 100 non‐IBS and 99 IBS individuals were analyzed.

The characteristics of non‐IBS and IBS individuals are shown in Table 1.

**TABLE 1:** Participant characteristics of non‐IBS and IBS groups.

Characteristic	Non‐IBS group (*n* = 100)	IBS group (*n* = 99)	*p*‐Value
IBS questionnaire for HCP of WGO (score)	6 (5–8)	25 (24–26)	<0.05^a^
Age (years)	24.5 (21–39)	41 (33–49)	<0.05^a^
Women/men (number)	50/50	58/41	0.224^b^
Weight (kg)	77.5 (59.75–89)	67 (61–83)	0.050^a^
Height (cm)	175 (167–186)	166 (164–182)	<0.05^a^
Body mass index (kg/m^2^)	23.6 ± 2.5	23.7 ± 2.0	0.6652^c^

*Note*: Data are expressed as mean ± standard deviation (SD) for normally distributed variables or median (interquartile range) for non‐normally distributed data or numbers.

^a^ Mann–Whitney *U* test.

^b^ Pearson's Chi‐squared test.

^c^ Student's *t*‐test.

Statistical analyses revealed no significant differences (*p* ≥ 0.05) between the two groups concerning the ratio between women and men, weight, and body mass index. The difference between the diagnostic scores determined with the IBS questionnaire for HCP of the two groups was statistically significant (*p* < 0.05). There were also statistically significant differences between the two groups in height and age. To account for the age difference, all analyses were also performed for an age‐matched non‐IBS group created by selecting 40 non‐IBS individuals with a comparable average age to the IBS group. Analyzing the data of the age‐matched non‐IBS group revealed no differences compared with analyses performed using the 100 individuals non‐IBS group (data not shown).

### 3.2 Emotional suppression scores of non‐IBS and IBS individuals

**FIGURE 2:**
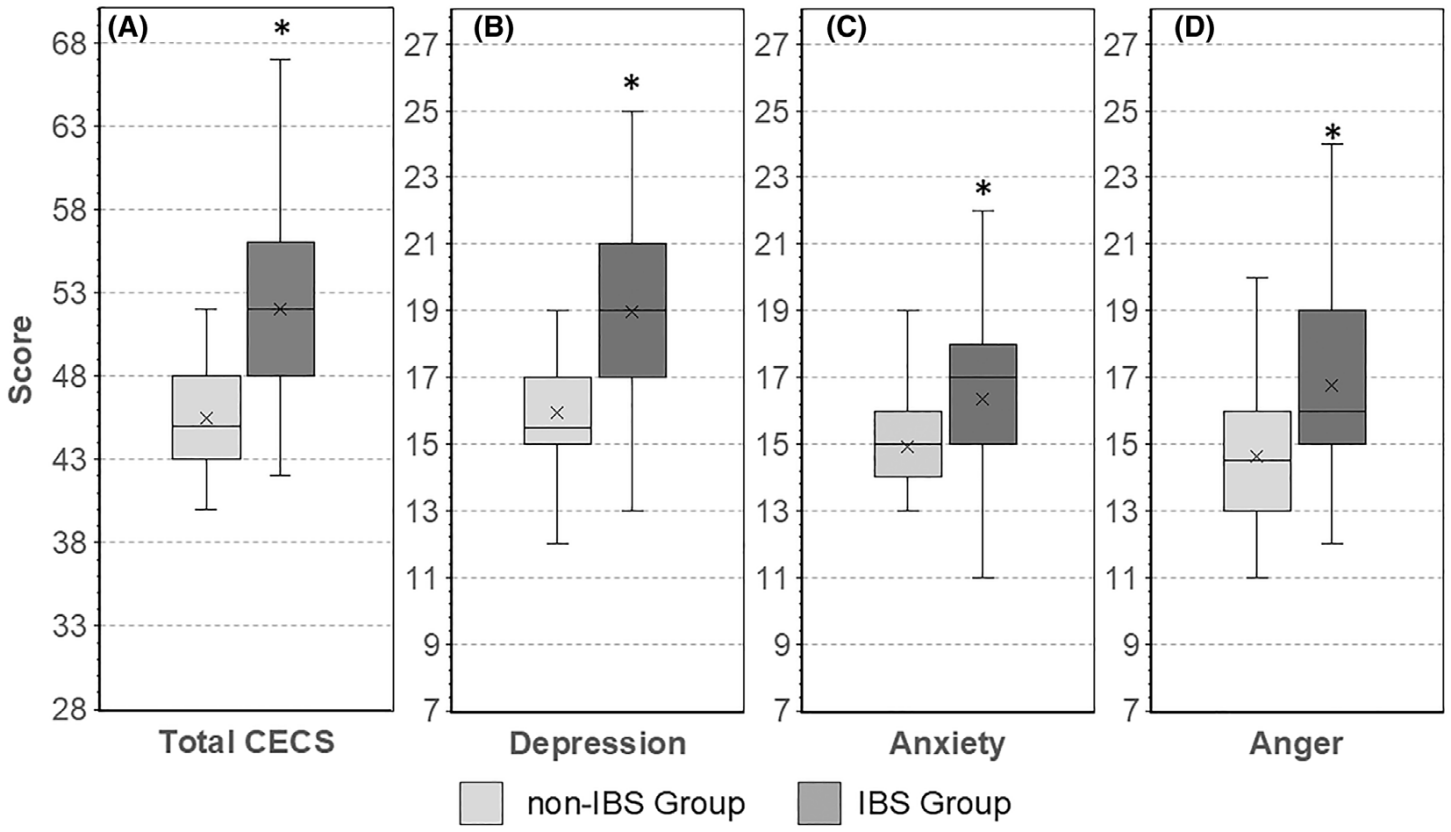
Level of emotional control assessed in non‐IBS and IBS individuals with the Courtauld Emotional Control Scale (CECS). (A) Total CECS scores, (B) depression subscale scores, (C) anxiety subscale scores, (D) anger subscale scores. The central line in the box plot represents the median, and the boxed areas represent the interquartile ranges. Differences in measurements between the non‐IBS and the IBS group were assessed with the Mann–Whitney *U* test, with asterisks indicating statistically significant (*p‐*value < 0.001) differences between the compared measurements.

Results obtained for the general coefficient of emotional control (total CECS) and the individual subscales for depression, anxiety, and anger are shown in Figure 2.

For the non‐IBS group, a general coefficient of emotional control of 45.0 (IQR 43–48) was determined. Emotional control of depression was highest (15.5 IQR 15–17), followed by suppression of anxiety (15.0 IQR 14–16) and that of anger (14.5 IQR 13–16). In the IBS‐ group, the general coefficient of emotional control was significantly (*p* < 0.001) higher (52.0 IQR 48–56) than in the non‐IBS group. The most suppressed emotion in IBS subjects was depression. Compared to that of non‐IBS subjects (15.5 IQR 15–17), the median depression subscale score of IBS subjects (19.0 IQR 17–21) was 22.6% higher (*p* < 0.001). The median anxiety subscale score of the IBS‐subject group was 13.3% higher than that of the non‐IBS group (17.0 IQR 15–18 vs. 15.0 IQR 14–16) (*p* < 0.001), while that of the anger subscale was 10.3% higher (16.0 IQR 15–19 vs. 14.5 IQR 13–16) (*p* < 0.001).

## 4 DISCUSSION

IBS is frequently associated with a wide range of neuropsychiatric symptoms, among them those of depression, anxiety, and anger. Elevated levels of depression, anxiety, and anger in IBS individuals compared with healthy controls have been reported in several clinical trials.^20^, ^23^, ^37^ Prevalence rates of up to 30% for depression and up to 40% for anxiety have been published.^13^, ^38^ The psychological stress adds to the burden of disease experienced by subjects suffering from IBS. A recently published systematic review has investigated the strategies used by IBS individuals to cope with disease‐associated psychological stress.^39^ The authors concluded that IBS individuals use a wide variety of coping strategies, including avoidance behavior as well as proactively addressing the issues at hand. The use of avoidance‐oriented coping by IBS individuals predicts a poor health outcome, while the currently available results regarding active‐coping strategies were found to be inconclusive. So far, little data have been published regarding the direct measurement of emotional suppression in IBS individuals. In a single‐case design study, it was found that emotional suppression might be an avoidance‐oriented coping strategy employed by IBS individuals; however, with data from only four individuals, the evidence is slim.^33^

So far, the present study is the largest controlled clinical observational trial measuring levels of emotional control in IBS subjects. The results obtained with the Courtauld Emotional Control Scale revealed a significantly higher general emotional control level in IBS individuals when compared with non‐IBS individuals. This shows that, similar to other illnesses, IBS is a condition where affected subjects tend to use emotional suppression in response to the psychological stress associated with the disease. In most situations, suppressing emotions is considered to have negative implications for individuals.^31^, ^32^ If this is also the case for IBS subjects, it will require further research.

Results from the present study indicate the need to increase the awareness of emotional suppression as part of the clinical picture of IBS. As emotional suppression seems to be a part of the clinical picture of IBS subjects, at least in individuals with moderate to severe forms of the disease, treatment should consider addressing the problem by making use of evidence‐based psychological interventions directed to reduce emotional suppression. Among the psychological interventions that could be considered are cognitive‐behavioral therapy (CBT) and gut‐directed hypnotherapy. For both approaches, evidence of efficacy in IBS subjects has been found.^40^, ^41^, ^42^, ^43^ Mindfulness‐based therapy has also been described to be effective in IBS; however, the evidence is limited, due to quality issues of the studies investigating the efficacy.^44^, ^45^, ^46^

## 5 STRENGTHS AND LIMITATIONS

The strength of the presented trial is its design as a controlled study recruiting IBS individuals diagnosed and assessed with a series of well‐established and validated tools (IBS questionnaire for HPC of the WGO; IBS‐SSS and CECS). The present study is the largest assessing emotional control in IBS individuals. The number of individuals participating in the study was large enough to determine statistically significant differences between the IBS group and the non‐IBS study participants.

The present study used a pragmatic approach, with little involvement of physicians and voluntary participation from individuals. This approach comes with several limitations. Assessing depression, anxiety, and anger with established and validated scales would have added tremendously to the value of the trial. However, this has been reserved for a later trial.

The most important limitation of the present study is the selection of individuals for the non‐IBS group. Individuals for the trial's control group were selected based on a “non‐IBS” diagnosis. While the study exclusion criteria avoided the enrollment of individuals with some diseases, it cannot be ruled out that at least some of the individuals in the non‐IBS group might suffer from other diseases. These diseases might be associated with some degree of personal emotional stress (depression, anxiety, or anger). Therefore, the emotional control levels determined for at least some of the non‐IBS group trial participants might be (at least to a certain degree) elevated compared with individuals free of somatic or psychological illnesses. Interestingly enough, the total CECS score determined for non‐IBS trial participants in the present trial (45.0 ± 5.0) was comparable to that (47.1 ± 5.0) found for a large (*n* = 362) group of healthy controls in a trial published in 2015.^29^

During the planning phase of the study, it was assumed that emotional suppression would be more common and more pronounced in more severe forms of the disease. Consequently, only moderate and severe forms of IBS were included in this pilot study. This decision may limit the generalizability of the findings and introduce a potential selection bias, two limitations that have to be considered when conclusions are drawn from the results of the study.

No additional validated instruments were used to exclude other disorders of the gut–brain interaction. This omission could result in diagnostic overlap and, therefore, represents a potential confounder.

The study has used the IBS questionnaire for HCP of the WGO for the diagnosis of IBS instead of the more widely accepted and standardized Rome IV criteria. This choice may limit the comparability with other studies.

Another limitation of the study is that the measurement was done only at one point in time, and any fluctuation of the level of emotional suppression over time was not investigated. Finally, the present study allows for the assessment of the presence of emotional suppression in IBS individuals, but it does not determine any causality.

## 6 CONCLUSIONS

Results from the present study show statistically significant elevated levels of emotional control in IBS patients compared with non‐IBS patients. Elevated suppression was found for depression, anxiety, anger, and total emotions. Concealing such feelings is generally not considered a constructive strategy for resolving psychological stress. Physicians and IBS subjects should be aware of this problem, and the management of IBS should consider including therapeutic approaches (e.g., psychological interventions like cognitive‐behavioral therapy or gut‐directed hypnotherapy) to address the problem of emotional control. Future clinical studies with IBS patients are needed to generate a more in‐depth understanding of emotional suppression in IBS patients. Future interventional clinical studies with IBS subjects should consider measuring emotional control levels in addition to evaluating effects on the gastrointestinal symptoms of IBS.

## AUTHOR CONTRIBUTIONS

**Henning Sommermeyer:** Conceptualization; validation; formal analysis; writing – original draft; writing – review and editing. **Magdalena Ciesla:** Conceptualization; methodology; data curation. **Dominika Szczerbiec:** Methodology; software; validation; formal analysis; data curation; writing – review and editing; visualization. **Pawel Olszewski:** Validation; investigation. **Paulina Wojtyla‐Buciora:** Investigation. **Jacek Piatek:** Conceptualization; methodology; investigation; resources; data curation; writing – review and editing; supervision; project administration.

## FUNDING INFORMATION

This research received no external funding.

## CONFLICT OF INTEREST STATEMENT

The authors have stated explicitly that there are no conflicts of interest in connection with this article.

## ETHICS STATEMENT

Ethics approval statement: The study was approved by the Ethics Committee of Calisia University (project identification code 1/2023, January 25, 2023).

Patient consent statement: All participants provided written informed consent.

Clinical trial registration: None.

## Data Availability

The data that support the findings of this study are available on request from the corresponding author. The data are not publicly available due to privacy or ethical restrictions.
